# Changes in SBPase activity influence photosynthetic capacity, growth, and tolerance to chilling stress in transgenic tomato plants

**DOI:** 10.1038/srep32741

**Published:** 2016-09-02

**Authors:** Fei Ding, Meiling Wang, Shuoxin Zhang, Xizhen Ai

**Affiliations:** 1 College of Forestry, Northwest A&F University, 3 Taicheng Rd., Yangling, Shaanxi 712100, China; 2 State Key Laboratory of Crop Biology, College of Horticulture Science and Engineering, Shandong Agricultural University, 61 Daizong St., Tai’an, Shandong 271018, China

## Abstract

Sedoheptulose-1, 7-bisphosphatase (SBPase) is an important enzyme involved in photosynthetic carbon fixation in the Calvin cycle. Here, we report the impact of changes in SBPase activity on photosynthesis, growth and development, and chilling tolerance in SBPase antisense and sense transgenic tomato (*Solanum lycopersicum*) plants. In transgenic plants with increased SBPase activity, photosynthetic rates were increased and in parallel an increase in sucrose and starch accumulation was evident. Total biomass and leaf area were increased in SBPase sense plants, while they were reduced in SBPase antisense plants compared with equivalent wild-type tomato plants. Under chilling stress, when compared with plants with decreased SBPase activity, tomato plants with increased SBPase activity were found to be more chilling tolerant as indicated by reduced electrolyte leakage, increased photosynthetic capacity, and elevated RuBP regeneration rate and quantum efficiency of photosystem II. Collectively, our data suggest that higher level of SBPase activity gives an advantage to photosynthesis, growth and chilling tolerance in tomato plants. This work also provides a case study that an individual enzyme in the Calvin cycle may serve as a useful target for genetic engineering to improve production and stress tolerance in crops.

The Calvin cycle is the primary pathway for photosynthetic carbon fixation in C_3_ plants and takes place in the stroma of chloroplasts. This cycle plays a key role in plant metabolism, providing intermediates required for starch, sucrose, and shikimic acid biosynthesis^1^. The Calvin cycle includes tree major steps: carboxylation of ribulose-1, 5-bisphosphate (RuBP), reduction of 3-phosphoglycerate, and regeneration of the CO_2_ acceptor RuBP. The enzyme sedoheptulose-1, 7-bisphosphatase (SBPase: EC 3.1.3.37) functions at the branch point where assimilated carbon may either go to the regenerative phase to produce the CO_2_ acceptor molecule RuBP in the cycle or be exported from the cycle for sucrose or transitory starch biosynthesis. This particular position highlights the importance of SBPase in regulating carbon flow in the Calvin cycle1^1^,^2^,^3^. Unlike other enzymes in the Calvin cycle, SBPase is unique to photosynthetic organisms where it catalyzes the dephosphorylation of sedoheptulose-1, 7-bisphosphate to sedoheptulose-7-phosphate. The activity of SBPase is highly regulated by a number of factors, such as pH and Mg^2+^^4^,^5^. In addition, like many Calvin cycle enzymes, SBPase is activated via ferredoxin/thioredoxin system in response to light^6^,^7^.

Modeling and metabolic control analyses have been made to investigate the importance of individual enzymes that control carbon flux through the Calvin cycle. Previous analysis of Rubisco has shown that more than 50% reduction in Rubisco level had little effects on photosynthesis for plants grown in moderate light and temperature conditions, though reductions in photosynthesis were proportionate to decreases in Rubisco activity under high light conditions^8^,^9^,^10^,^11^. A large proportion of reductions in the levels of glyceraldehyde-3-P dehydrogenase, Fru-1, 6-bisphosphatase (FBPase) and phosphoribulokinase (PRKase) is not limiting photosynthetic capacity through the cycle^10^,^11^,^12^,^13^, implying that these individual enzymes exist in excess and are not at the levels of limiting carbon fixation through the Calvin cycle. In contrast, small reductions in SBPase activity lead to significant decreases in photosynthesis in SBPase antisense tobacco plants^14^,^15^,^16^, providing direct evidence that SBPase is tightly linked with carbon flux in the Calvin cycle. Moreover, transgenic tobacco plants with increased SBPase activity exhibit higher levels of sucrose and starch accumulation and increased leaf area and biomass^17^,^18^. Later work has shown that overexpression of SBPase improves photosynthetic carbon gain and yield in tobacco plants grown under field conditions^19^. A more recent study has demonstrated that the mutation of *SBPase* severely retards growth and development through inhibiting carbon assimilation efficiency in *Arabidopsis*^20^. Additionally, overexpression of SBPase protects transgenic rice against salt stress and high temperature stress by providing more regeneration of the CO_2_ acceptor molecule RuBP in the stroma^21^,^22^. All these lines of evidence suggest that SBPase plays a critical role in control over carbon flow in the Calvin cycle.

Tomato is one of the most important horticultural crops in the world. Due to its tropical/subtropical origin and domestication, tomato plants are sensitive to chilling. When exposed to temperatures below 10 °C, tomato plants generally suffer chilling injury, and extended exposure to temperatures below 6 °C often severely damages tomato plants^23^. Chilling is thus considered as a factor limiting growth and yield in tomato production. Genetically engineering tomato plants with a suitable target gene offers potential for the amelioration of chilling injury. Much work on SBPase has been done in tobacco and *Arabidopsis*; however, information on SBPase in vegetable crops, e.g. tomato, is rather limited. To our knowledge, the role of SBPase in protection of plants against chilling stress has not yet been reported. The objectives of our present work were (1) to assess the impact of changes in SBPase activity on photosynthetic capacity and growth in tomato plants and (2) to explore the protective role of increased SBPase activity in tomato plants exposed to chilling stress. This work provides a case study that an individual enzyme in the Calvin cycle is a useful target for genetic engineering to improve production and stress tolerance in horticultural plants.

## Results

### Isolation of tomato *SBPase* gene

**Figure 1:**
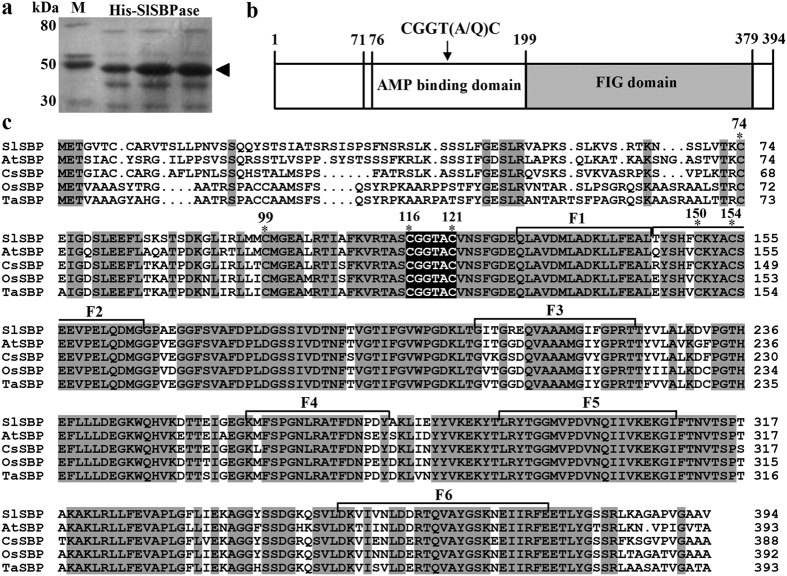
A schematic diagram illustrating the functional domains of SlSBPase and multiple alignments of SlSBPase-related proteins. (**a**) SDS-PAGE of SlSBPase protein. Arrows indicate the HIS-SlSBPase protein. (**b**) Structure of SlSBPase. (**c**) Alignment of deduced amino acid sequences of SBPase from five plant species. Identical amino acid residues are shown on grey backgrounds. The amino acid residues showing the regions involved in redox-regulated activation of SBPase are boxed. F1–F6 amino acid residues are six SBPase fingerprints. The accession numbers of SBPase in GenBank are as follows: *Solanum lycopersicum*, FJ959073; *Arabidopsis thaliana*, AEE79443; *Cucumis sativus*, ACQ82818; *Oryza sativa*, AAO22558; *Triticum aestivum*, CAA46507.

A query of the tomato genome database with the deduced amino acid sequence of *Arabidopsis* SBPase (At3g55800) using BLAST revealed a sequence (Solyc05g02600) in the tomato genome that had 81% amino acid sequence homology to *Arabidopsis* SBPase, and the sequence was referred to hereafter as *SlSBPase*. The full-length cDNA of *SlSBPase* is 1564 bp, containing a coding region of 1185 bp. Sequence analysis indicated that *SlSBPase* encodes a polypeptide of 394 amino acids. Prokaryotic expression analysis revealed that His-SlSBPase fusion protein was about 48 kDa, in agreement with the predicted SlSBPase size of 42.6 kDa (Fig. 1a). Two domains, AMP binding domain and FIG (FBPase/IMPase/glpX) domain, were found in tomato SBPase (Fig. 1b), and these two domains are also present in rice and cyanobacteria FBP/SBPase^24^,^25^. Sequence alignment analysis revealed that SlSBPase shared 81%, 84%, 79% and 78% homology to SBPase amino acid sequences of *Arabidopsis thaliana*, *Cucumis sativus*, *Oryza sativa* and *Triticum aestivum*, respectively (Fig. 1c), indicating that the evolution of SBPase is highly conservative. In addition, six sedoheptulose-1, 7-bisphosphatase fingerprints (F1–F6) were found in tomato SBPase (Fig. 1c). The motif CGGTAC existing in redox-active groups of a number of enzymes has also been observed in SlSBPase. Cys116 and Cys121 in this motif are predicted to be involved in redox-regulated activation of tomato SBPase as observed in wheat^26^, rice^27^, cucumber^28^ and *Arabidopsis*^15^.

### *SlSBPase* expression pattern and SBPase activity in tomato plants

**Figure 2:**
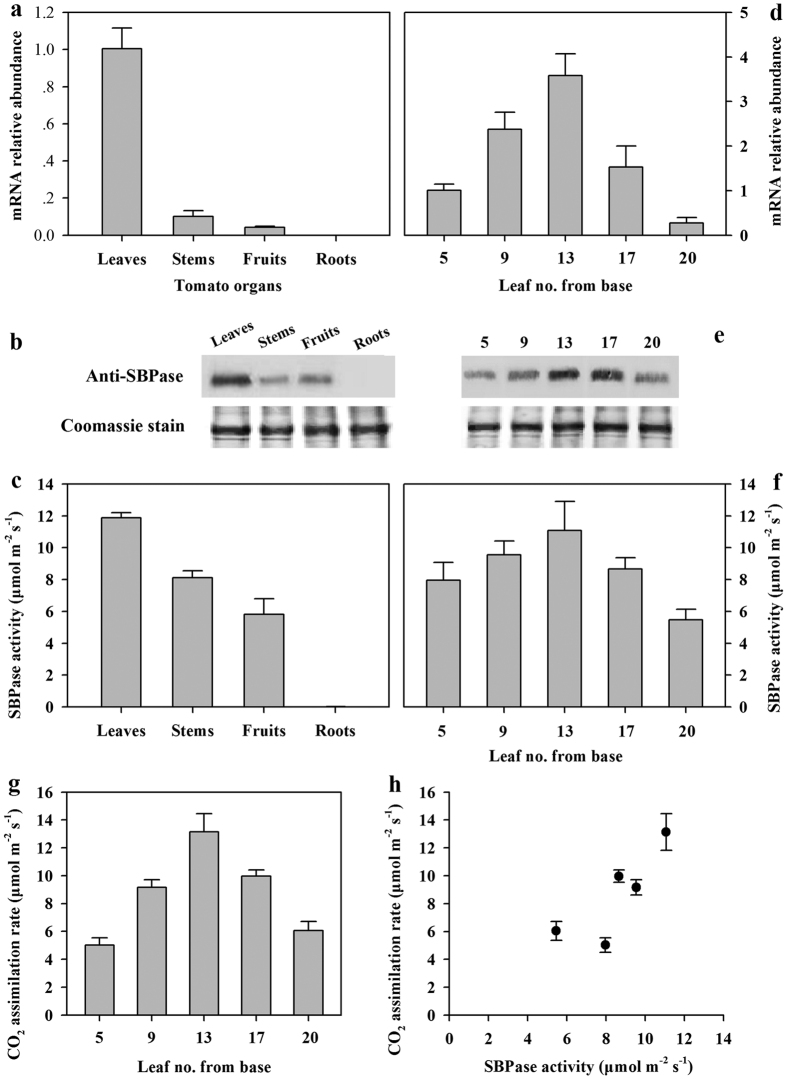
*SlSBPase* mRNA abundance, SBPase activity and protein levels in different tissues and during leaf development in tomato plants. All measurements were done on 20-leaf tomato plants. (**a**,**d**) *SlSBPase* mRNA abundance was measured by quantitative real-time PCR using total RNA separately isolated from different organs (fully expanded leaves, one-week old fruits, stems, roots) and leaves at different developmental stages including post-maturation leaves (leaf no. 5 from base), mature fully expanded leaves (leaf no. 9, 13), new fully expanded leaves (leaf no. 17) and young expanding leaves (leaf no. 20) and in tomato plants. (**b**,**e**) Protein levels. 25 μg protein samples from different tissues were separated by SDS-PAGE. SBPase protein was stained by coomassie blue and was subjected to western blot analysis with an anti-SBPase polyclonal antibody. (**c**,**f**) SBPase activity. The same tissues for *SlSBPase* mRNA analysis were sampled for SBPase activity assay. (**g**) CO_2_ assimilation rate in leaves at the different developmental stages. (**h**) CO_2_ assimilation rate as a function of SBPase activity. The results are the means ± SDs (n = 4).

SBPase is a key enzyme in the Calvin cycle, so the first goal in our study was to investigate the expression pattern of *SlSBPase* in tomato plants through quantitative real-time PCR analysis. The results showed that *SlSBPase* was expressed in leaves, stems and fruits, but not in roots (Fig. 2a). Western blots with an anti-SBPase polyclonal antibody revealed the presence of a strong positive protein signal corresponding to SlSBPase in leaves, weak signals in stems and fruits, but no signal in roots (Fig. 2b). Consistent with the expression pattern of *SlSBPase*, SBPase activity was found to be the highest in leaves, but barely detected in roots (Fig. 2c). All these results indicate that *SlSBPase* was expressed only in green tissues, in keeping with its biological functions in the chloroplast.

It has been reported that changes of photosynthetic capacity are dependent on the stages of leaf development^29^. To explore the relationship between SBPase activity and photosynthesis, we examined the *SlSBPase* expression pattern, SBPase activity and photosynthesis during leaf expansion and maturation. *SlSBPase* transcripts were found more in mature fully expanded leaves (leaf no. 9, 13 from base) and new fully expanded leaves (leaf no. 17) than in post-maturation leaves (leaf no. 5) and young expanding leaves (leaf no. 20, Fig. 2d). This developmental expression pattern was also confirmed by western blot analysis (Fig. 2e). Similar patterns were also found for SBPase activity (Fig. 2f) and photosynthesis (Fig. 2g). Further analysis showed that changes in photosynthetic rate showed relationship with the changes in SBPase activity (Fig. 2h). These results suggest that SBPase might be involved in the development-dependent changes in photosynthetic rate.

### Diurnal dynamics of *SlSBPase* expression, SBPase activity and photosynthesis in tomato plants grown in greenhouse

**Figure 3:**
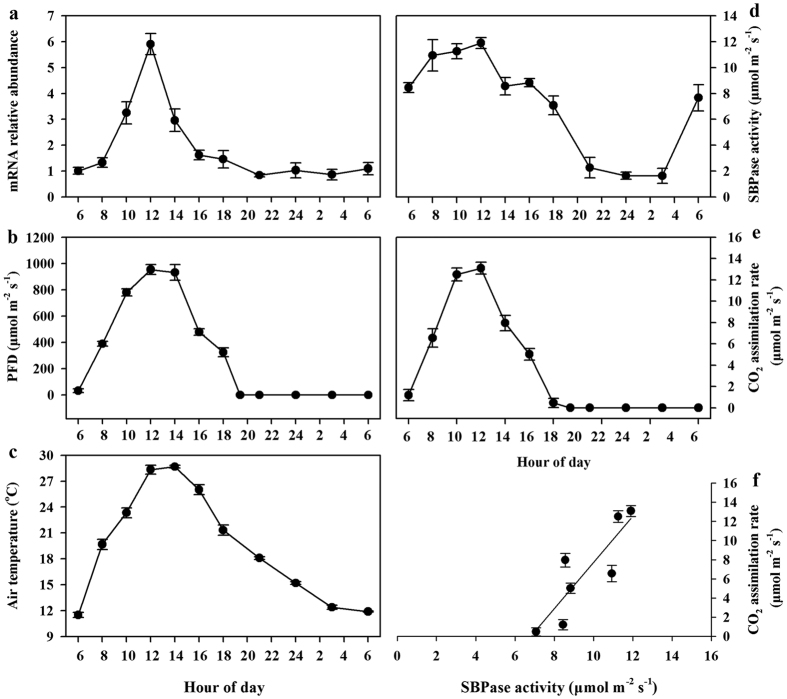
Diurnal changes in *SlSBPase* mRNA abundance, SBPase activity, CO_2_ assimilation rate, PFD and air temperature measured in greenhouse conditions on three consecutive sunny days. All measurements were done on 20-leaf tomato plants. (**a**) *SlSBPase* mRNA abundance was analyzed by quantitative real-time PCR using total RNA isolated from the fully expanded leaves (leaf no. 13–15 from base) at 2-h intervals. (**b**) Diurnal variation of PFD in greenhouse. (**c**) Diurnal variation of air temperature in greenhouse. (**d**) SBPase activity was assayed using the same tissues for *SlSBPase* mRNA analysis. (**e**) Measurements of CO_2_ assimilation rate were made on the fully expanded leaves (leaf no. 13–15) at 2-h intervals. (**f**) CO_2_ assimilation rates were plotted against levels of SBPase activity, showing the correlation between them. The results are the means ± SDs (n = 4).

To gain further understanding of relationship between SBPase activity and photosynthesis, we monitored the fluctuations of *SlSBPase* expression, SBPase activity and photosynthesis on a 24-h scale under greenhouse conditions. At the 20-leaf stage, all measurements were performed on fully expanded leaves (leaf no. 13–15 from base) on a sunny day. The mRNA abundance of *SlSBPase* rose within a relatively short time with the increasing photon flux density (PFD) and air temperature (Ta) in the morning, and reached the maximum around 12:00 (Fig. 3a–c). Subsequently, the mRNA abundance of *SlSBPase* decreased, and stayed at a low level throughout the night. SBPase activity also peaked around 12:00, however, unlike the sharp increase in mRNA abundance of *SlSBPase*, a gentle increase in SBPase activity was observed between 8:00 and 12:00 (Fig. 3d). The discrepancy is probably due to the biochemical process from mRNA to functional protein and the highly regulatory property of SBPase. During the dark period, only a minimum of SBPase activity was observed, which was possibly due to low expression level of *SlSBPase* at night and lack of light activation of SBPase enzyme activity. It has been reported that the proportion of active SBPase can be induced by more than 10-fold in response to light^6^,^7^.

Diurnal dynamics of photosynthesis followed a single-peak curve in tomato plants, with the highest photosynthetic rate occurring at 12:00 (Fig. 3e), consistent with changes of *SlSBPase* expression and SBPase activity. Combined with a previous report that small changes in SBPase activity alter photosynthesis in transgenic tobacco plants^16^, we hypothesized that changes in the levels of SBPase activity contributed to altered photosynthesis in tomato plants. To test our hypothesis, we performed a preliminary analysis by plotting photosynthesis against SBPase activity. SBPase activity was linearly correlated (R^2^ = 0.75) with photosynthesis in tomato plants (Fig. 3f). In the following experiments, we produced SBPase sense and antisense transgenic tomato plants with a wide arrange of SBPase activities to further examine SBPase-photosynthesis relationship.

### Changes in SBPase activity affected photosynthetic capacity in transgenic tomato plants

**Figure 4:**
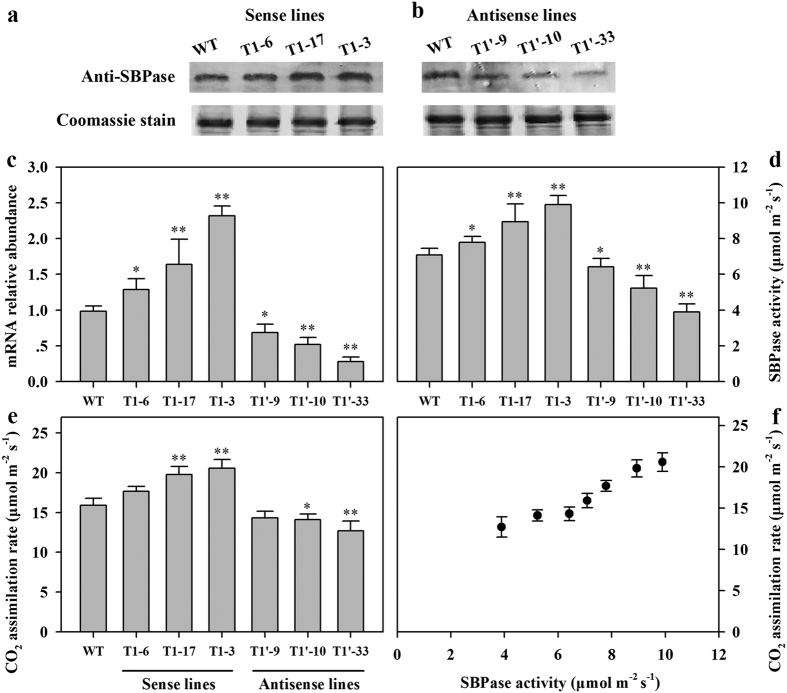
*SlSBPase* mRNA abundance, SBPae protein level, SBPase activity and CO_2_ assimilation rate in different transgenic lines. All measurements were done on 12-leaf wild-type and T1 progeny of transgenic tomato plants. (**a**,**b**) Protein levels. 25 μg protein samples from the fully expanded leaves (leaf no. 8, 9 from base) in wild-type and transgenic tomato plants were separated by SDS-PAGE. SBPase protein was stained by coomassie blue and was subjected to western blot analysis with an anti-SBPase polyclonal antibody. (**c**) *SlSBPase* mRNA abundance was analyzed by quantitative real-time PCR using total RNA isolated from the fully expanded leaves (leaf no. 8, 9). (**d**) SBPase activity was assayed using the same tissues for *SlSBPase* mRNA analysis. (**e**) CO_2_ assimilation rate measurements were made on the fully expanded leaves (leaf no. 8, 9) in greenhouse conditions. (**f**) Effect of changes in SBPase activity on CO_2_ assimilation rate under greenhouse conditions. The results are the means ± SDs (n = 5). Asterisks indicate significant differences between WT and transgenic plants (* for *P* < 0.05; ** for *P* < 0.01).

To gain further insights into the relationship between SBPase activity and photosynthesis, we generated SBPase sense and antisense tomato plants. Following transformation, 25 primary transformants (T0 generation) with *SBPase* sense constructs and 29 primary transformants with *SBPase* antisense constructs were obtained. The resulting seeds from primary transformants were sown and plants of T1 progeny were used for further examination in the following experiments. T1 transgenic plants with different expression levels of *SlSBPase* were selected by quantitative real-time PCR analysis and western-blot analysis (Fig. 4a–c). Quantitative real-time PCR analysis of selected transgenic lines confirmed no significant changes in the expression level of other enzyme-coding genes in the Calvin cycle (see Supplementary Fig. S1). Enzyme activity assay confirmed three lines of SBPase sense plants with increased activities of SBPase and three lines of SBPase antisense plants with decreased activities of SBPase (Fig. 4d). Together, this cohort of plants possesses a wide range of SBPase activities, allowing us to closely examine how SBPase activity might affect photosynthesis in tomato plants.

Gas exchange measurements were made to investigate whether changes in SBPase activity influence changes in carbon assimilation rates in transgenic tomato plants under greenhouse conditions. Most of previous studies showing that SBPase affects photosynthesis have been done in tightly controlled environment, with the exception of a study that the SBPase overexpressing lines had slightly higher rates of photosynthesis and accumulated more fixed carbon than wild-type plants over a day under greenhouse conditions^17^. It is interesting and of practical significance to understand how changes in SBPase activity in tomato influence photosynthesis and growth in greenhouse environment in which tomato production commonly takes place. Hence, measurements of photosynthetic activity were performed under greenhouse conditions in our study. CO_2_ assimilation rates were determined on the fully expanded leaves (leaf no. 8, 9 from base) of 12-leaf wild-type and transgenic tomato plants at 10:30–11:30 a.m. on a sunny day. Photosynthetic rate and SBPase activity were highest in SBPase sense transgenic line T1-3, in contrast, the lowest photosynthetic rate with the lowest SBPase activity were observed in SBPase antisense transgenic line T1′-33 (Fig. 4e). Plotting photosynthetic rates against SBPase activities revealed a pattern that photosynthetic rate increased as SBPase activity went higher (Fig. 4f), in concert with the relationship we observed between SBPase activity and photosynthesis in our experiment on development-dependent changes in photosynthesis.

### Changes in SBPase activity affected carbohydrate levels in transgenic tomato plants

**Figure 5:**
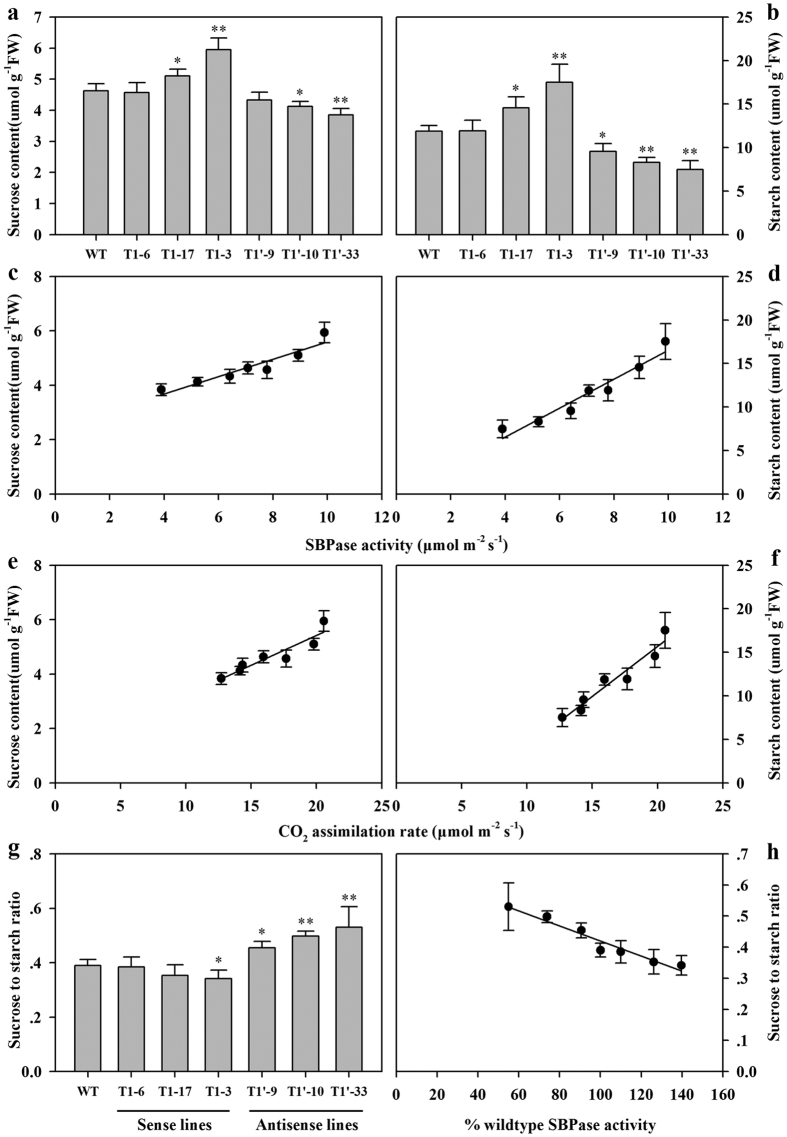
Carbohydrate content in fully expanded leaves of SBPase sense and antisense tomato plants. All measurements were done on 12-leaf wild-type and T1 progeny of transgenic tomato plants. (**a**,**b**) Contents of sucrose and starch were determined in fully expanded leaves (leaf no. 8, 9 from base) harvested at the end of day. (**c**) Sucrose level as a function of SBPase activity. (**d**) Starch content as a function of SBPase activity. (**e**) Sucrose content as a function of CO_2_ assimilation rate. (**f**) Starch content as a function of CO_2_ assimilation rate. (**g**) Sucrose-to-starch ratios in SBPase sense and antisense tomato plants. The results are the means ± SDs (n = 6). Asterisks indicate significant differences between WT and transgenic plants (* for *P* < 0.05; ** for *P* < 0.01).

To study the impact of SBPase activity on carbohydrate accumulation, we examined the content of sucrose and starch at the end of day in wild-type and transgenic plants. In both wild-type plants and transgenic plants, sucrose and starch accumulated during the light period. Transgenic line T1-3 with the highest SBPase activity accumulated up 28% more sucrose and 47% more starch than equivalent wild-type plants, while antisense transgenic line T1′-33 with the lowest SBPase activity accumulated significantly less sucrose and starch than wild-type plants (*P* < 0.01) (Fig. 5a,b). A linear correlation (R^2^ = 0.88 for sucrose; R^2^ = 0.93 for starch) between increased SBPase activity and increased carbohydrate accumulation was evident, in agreement with the importance of SBPase in carbon assimilation. It is noted that at higher levels of SBPase activity, the levels of sucrose and starch varied dramatically over a narrow of SBPase activity (Fig. 5c,d), suggesting that carbohydrate status is more sensitive to high levels of SBPase activity. The relationship between photosynthetic rates and carbohydrate levels were also examined. It was shown that the accumulation of sucrose and starch was increased as photosynthetic rates increased and the increases were more pronounced at high levels of photosynthetic rate (Fig. 5e,f), which indicates that difference in photosynthetic capacity, at least partially, accounted for the changes in carbohydrate levels. The sucrose-to-starch ratio was also changed and the change appeared more dramatic in antisense plants than sense plants (Fig. 5g,h). In antisense tomato plants, as the levels of SBPase activity decreased, the sucrose-to-starch ratio became higher, showing a shift in carbon allocation towards soluble carbohydrates. Similar change of sucrose-to-starch ratio has also been reported in SBPase antisense tobacco plants^14^.

### Effects of changes in SBPase activity on growth and development in transgenic tomato plants

**Figure 6:**
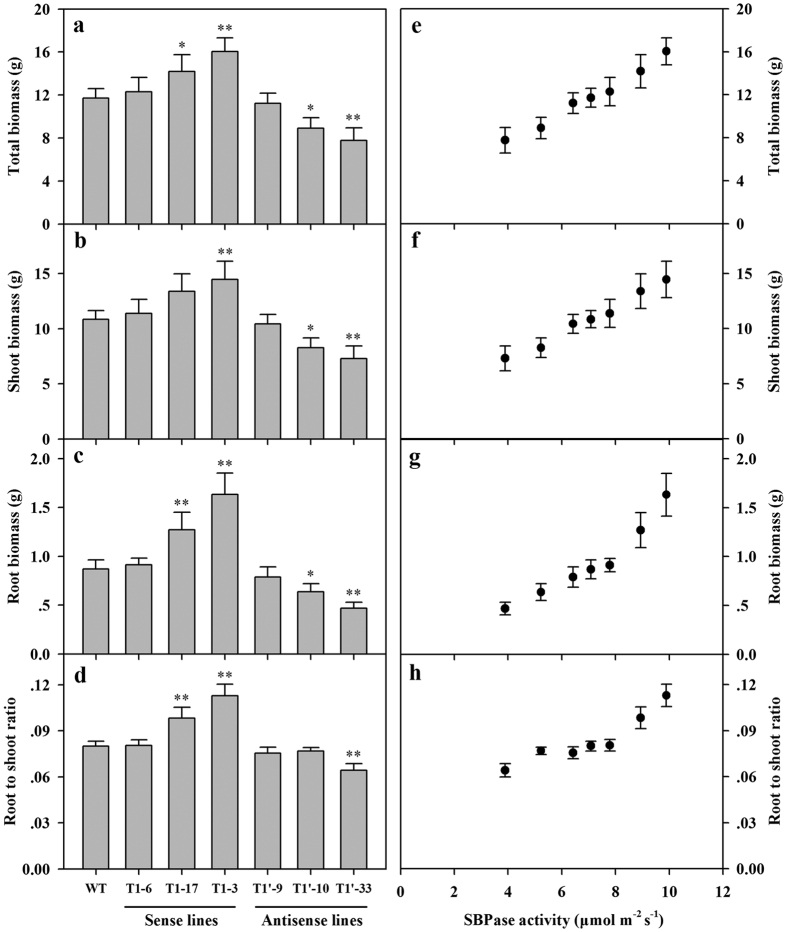
Biomass of SBPase sense and antisense tomato plants. All measurements were done on 12-leaf wild-type and T1 progeny of transgenic tomato plants. (**a**) Total plant biomass. (**b**) Shoot biomass. (**c**) Root biomass. (**d**) Root/shoot ratio. (**e**) Total biomass as a function of SBPase activity. (**f**) Shoot biomass as a function of SBPase activity. (**g**) Root biomass as a function of SBPase activity. (**h**) Root to shoot ratio as a function of SBPase activity. The results are the means ± SDs (n = 6). Asterisks indicate significant differences between WT and transgenic plants (* for *P* < 0.05; ** for *P* < 0.01).

To obtain data on biomass of tomato plants, SBPase sense and antisense plants were grown in greenhouse conditions. At the 12-leaf stage, wild-type and transgenic plants were harvested and the dry weight of roots and shoots were determined. Differences in growth rate and biomass production were evident between transgenic plants and wild-type plants. Total biomass was increased in response to increase in SBPase activity in SBPase sense plants, while total biomass was decreased as SBPase activity was reduced in SBPase antisense plants (Fig. 6a). Further examination showed that the increase or decrease in total biomass was due to changes in both the shoot and root biomass (Fig. 6b,c) and a close relationship between SBPase activity and shoot biomass and root biomass was evident (Fig. 6e–g). The root-to-shoot ratio is one measure used to assess the overall health of plants. It also reflects the size of root sink in plants. Our results showed that SBPase sense plants had higher root-to-shoot ratio than SBPase antisense plants (Fig. 6d), implying that higher SBPase activity promoted root growth. We also noticed that at higher levels of SBPase activity (above 8 μmol m^−2^ s^−1^), increased SBPase activity improved root-to-shoot ratio, whereas at low levels of SBPase activity (below 8 μmol m^−2^ s^−1^), SBPase had less effect on root-to-shoot ratio (Fig. 6h), demonstrating that high SBPase activity favors root growth.

Transgenic tomato plants grown under greenhouse conditions were also used to investigate the impacts of different levels of SBPase activity on plant height, stem diameter, leaf morphology and flowering time. Measurements of plant height revealed that reductions in SBPase activity led to decreased height in antisense transgenic plants, however, an increase in SBPase activity did not change plant height much in SBPase sense transgenic plants (Table 1). Stem diameter was greater in SBPase sense plants than in antisense plants. Altered levels of SBPase activity also impacted leaf morphology by affecting leaf length and leaf width as well as length-to-width ratio. In general, as SBPase activity increased in transgenic plants, leaf length and leaf width increased. A close examination showed leaf width was more affected by SBPase activity than leaf length, resulting in wider leaves as was indicated by lower length-to-width ratio in plants with higher levels of SBPase activity (Table 1). The impact of SBPase activity on flowering time was also investigated in transgenic plants and wild-type plants. Flowering time was delayed 4–6 days in antisense plants compared with wild-type plants, in contrast, it was 4–7 days earlier in sense plants in which the levels of SBPase activity were high (Table 1). All these results illustrate a role of SBPase in plant growth and development.

### Higher levels of SBPase activity improved chilling tolerance in transgenic tomato plants

Previous studies have revealed that overexpression of SBPase improves carbon assimilation rates and growth in rice under salt stress and high temperature stress^21^,^22^. However, no information is available on how SBPase activity may affect plants under chilling stress. To investigate the possible role of SBPase in chilling tolerance in tomato plants, we grew a group of transgenic plants exhibiting higher and lower SBPase activities compared with wild-type plants in greenhouse, then these plants were acclimated in a growth chamber at 25 °C for 7 d. Following acclimation, tomato plants were exposed to 5 °C in growth chamber for 12 h.

**Figure 7:**
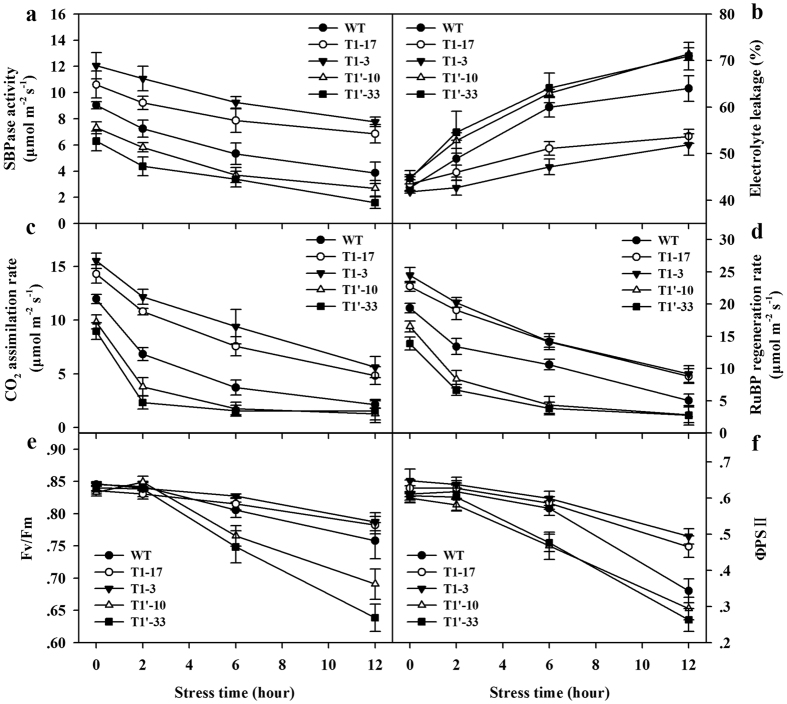
Effects of changes in SBPase activity on chilling tolerance in transgenic tomato plants. All measurements were done on 12-leaf wild-type and T1 progeny of transgenic tomato plants subjected to chilling stress at 5 °C for 12 h. (**a**) Changes in SBPase activity in response to chilling stress in transgenic plants. (**b**) Changes in electrolyte leakage in response to chilling stress in transgenic plants. (**c**) Changes in CO_2_ assimilation rates in response to chilling stress in transgenic plants. (**d**) Changes in RuBP regeneration rate in response to chilling stress in transgenic plants. (**e**,**f**) Changes in the maximum potential quantum efficiency (Fv/Fm) and the actual quantum efficiency (ΦPSII) of photosystem II in response to chilling stress in transgenic plants. The results are the means ± SDs (n > 4).

SBPase activity is highly regulated by a variety of factors, such as light, stromal Mg^2+^ concentration and pH level^4^,^5^,^7^,^30^. SBPase was also found to be sensitive to temperature changes in our experiment. Exposure to 5 °C substantially reduced the levels of SBPase activity in both wild-type plants and transgenic plants. Following 12 h of chilling treatment, SBPase activity was decreased by 57% in wild-type plants and the reduction in SBPase activity in antisense plants was even greater. The levels of SBPase activity in sense plants remained relatively higher than those in antisense plants and wild-type plants after a period of 12 h chilling (Fig. 7a).

Electrolyte leakage has been effectively used to determine tolerance to freezing/chilling in plants^31^,^32^. In our experiment, electrolyte leakage was increased in both wild-type plants and transgenic tomato plants (Fig. 7b). The increases in electrolyte leakage were greater in wild-type plants and SBPase antisense plants than those in SBPase sense plants. After 12 h exposure to chilling, electrolyte leakage was about 19% higher in antisense plants than in sense plants, indicating that increased SBPase activity might be involved in the mitigation of chilling-induced damage in tomato plants.

Photosynthetic rates were measured to assess the effects of changes in SBPase activity on chilling tolerance in tomato plants. Exposure to chilling for just 2 h considerably decreased photosynthetic rates in all tomato plants, however, the decreases were much less in SBPase sense plants than in wild-type plants and SBPase antisense plants (Fig. 7c). As chilling lasted, further decreases in photosynthesis were observed in all plants, but photosynthetic rates in SBPase sense plants remained relatively higher. In parallel with the decreases in photosynthetic rate, chilling stress induced great reductions in RuBP regeneration rate in all plants, however, a relatively higher RuBP regeneration rate was maintained in SBPase sense plants with higher levels of SBPase activity (Fig. 7d). These results indicate that changes in SBPase activity may affect photosynthetic capacity by altering the capacity for RuBP regeneration in tomato plants, consistent with a previous report that decreases in SBPase activity in transgenic tobacco plants limit carbon assimilation by reducing the capacity for RuBP regeneration^33^. Fv/Fm and ΦPSII represent the maximum potential quantum efficiency and the actual quantum efficiency of photosystem II, respectively. During chilling stress, the gradual decline in Fv/Fm and ΦPSII was observed occurring in both wild-type plants and transgenic plants, however, the decline was more pronounced in SBPase antisense plants (Fig. 7e,f). Thus, it is likely that in addition to increased RuBP regeneration, higher Fv/Fm and ΦPSII in SBPase sense plants with elevated levels of SBPase activity may also contribute to the relatively higher photosynthetic capacity under chilling stress.

## Discussion

The purpose of present work was to use transgenic tomato plants displaying a wide range of SBPase activities (55–139% of wild-type level) to investigate the impact of changes in SBPase activity on photosynthetic capacity, growth, and tolerance to chilling stress. Our findings are that (1) increased levels of SBPase activity promote photosynthetic activity; (2) different levels of SBPase affect growth and development in tomato plants by altering photosynthetic efficiency and carbohydrate status; (3) higher levels of SBPase activity confer improved chilling tolerance by accumulating more carbohydrate and maintaining relatively higher RuBP regeneration rates and quantum efficiency of photosystem II in tomato plants.

The levels of *SlSBPase* expression and SBPase activity varied dependent on the stages of leaf development in tomato plants, with the highest being in mature fully expanded leaves and the lowest being in young expanding leaves and post-maturity leaves (Fig. 2). This pattern of *SlSBPase* expression and SBPase activity is consistent with the pattern of photosynthetic rates during leaf development, reflecting the importance of SBPase in the demand for photosynthate as leaves transit from net carbon importers (young expanding leaves) to net carbon exporters (fully expanded optimal functional leaves). These results were in agreement with a previous study in which development-dependent changes in photosynthetic rate in relation with the levels of SBPase activity were observed in tobacco^16^. The diurnal dynamics of photosynthetic rates and levels of SBPase further support that SBPase is involved in the regulation of carbon assimilation in tomato plants. Another piece of evidence in favor of SBPase influencing photosynthetic capacity in tomato plants comes from our transgenic analysis. Photosynthetic rates were found increasing and decreasing in response to elevated levels of SBPase activity and reduced levels of SBPase activity, respectively. The transgenic data have clearly demonstrated a correlation between SBPase activity and photosynthetic rate (Fig. 4f, R^2^ = 0.92), in accordance with a previous study that photosynthetic capacity was compromised due to the carbonylation-induced reductions in SBPase activity in *Arabidopsis*^20^. In our study, a relatively small reduction in SBPase activity could cause an obvious decrease in photosynthetic rate in SBPase antisense tomato plants. In contrast to our findings, 65–90% reductions in the levels of the Calvin cycle enzymes, including Rubisco^8^, FBPase^12^, GAPDH^13^ and PRKase^34^ are required before bringing about any effect on photosynthesis. All these lines of evidence from our study and previous studies fit well with the key role of SBPase in the Calvin cycle and highlight SBPase as an enzyme exerting considerable control over carbon flux in tomato plants.

Analysis of changes in carbohydrate accumulation revealed that the levels of both sucrose and starch increased in SBPase sense plants and decreased in SBPase antisense plants in comparison with wild-type plants (Fig. 5). The increase in carbohydrate levels in response to increased SBPase activities may contribute to improved growth in tomato plants overexpressing SBPase. In support of this suggestion, we measured shoot biomass and root biomass in tomato plants and found that both shoot biomass and root biomass were increased in SBPase sense plants which accumulated more carbohydrates. In keeping with these results is a prior analysis of carbohydrate levels and leaf biomass in SBPase sense tobacco plants, which has revealed the correlation between carbohydrate accumulation and biomass^17^. In addition, we found that the sucrose-to-starch ratio increased in response to declined levels of SBPase activity and the increase was particularly apparent in SBPase antisense plants, which clearly indicates that the priority for tomato plants with suppressed SBPase activity is to maintain sucrose biosynthesis in favor of growth and obviously this is at the expense of starch. Similar changes in carbon allocation were also observed in FBPase and PRKase anstisense plants^12^,^34^. The decline in starch levels may result from the low levels of phosphoglycerate which deactivate ADP-glucose pyrophosphorylase, an important regulatory enzyme in starch biosynthesis, in the severely affected Calvin cycle in antisense tomato plants.

In agreement with the decreased levels of SBPase activity, it is noted that leaf size was much reduced in SBPase antisense tomato plants. The leaf width and length of antisense plants were significantly reduced compared with those of wild-type plants (*P* < 0.05) (Table 1), indicating that SBPase plays an important role in organ growth. These results could be validated by a previous observation that mutation in SBPase caused the inhibition of cell enlargement and the arrest of cell division, leading to leaf growth defects^20^. Changes in SBPase activity were also found to have an impact on reproductive development. The flowering occurred 13 d later in antisense tomato plants with the lowest SBPase activity than in sense plants with the highest SBPase activity, clearly indicating that reductions in SBPase activity perturbed the transition to flowering in tomato plants. One possible explanation for this delay in flowering is the much reduced accumulation of carbohydrate in SBPase antisense plants compared with sense plants. Previous studies have demonstrated that a certain level of carbohydrate supplied to shoot apex is required for the normal switch from vegetative to reproductive phase, which determines the onset of flowering^35^,^36^. These transgenic analyses have clearly shown a role of SBPase in plant growth and development.

Being originated and domesticated in tropical and subtropical regions, tomato plants are sensitive to chilling injury during all developmental stages, including seed germination, vegetative growth and reproductive phase. Therefore, chilling is an environmental factor limiting growth and yield in tomato production^23^,^37^,^38^,^39^. We found a dramatic decrease in SBPase activity in tomato plants exposed to 5 °C of chilling. In parallel, reduced photosynthesis was also observed. Moreover, in response to 12-h chilling stress, tomato plants showed a surge in electrolyte leakage, indicating severe disruption of cell membranes. All these results reveal the damages that chilling stress caused in tomato plants, however, as expected, chilling-induced damages could be alleviated by increased levels of SBPase, which was witnessed by increased carbon assimilation rates and decreased electrolyte leakage in transgenic tomato plants overexpressing SBPase. Recently, SBPase has been identified as a susceptible target enzyme to oxidative stress^20^. Thus, it can be speculated that under chilling stress, reactive oxygen species accumulated in tomato plants and induced the oxidative modification of SBPase, leading to reduced levels of SBPase activity. This loss of SBPase could be partially compensated for by overexpressing SBPase in tomato plants. After exposure to chilling for 12 h, SBPase sense plants maintained higher levels of SBPase activity than SBPase antisense plants and wild-type plants (Fig. 7). Furthermore, RuBP regeneration rates were found to be higher in sense tomato plants than those in antisense and wild-type plants during the period of chilling. This enhancement of RuBP regeneration may contribute to the improved photosynthesis in sense tomato plants under chilling conditions. ΦPSII is an indicator of quantum efficiency of electron transport through PSII. In our study, in response to chilling, a gradual decline in ΦPSII was observed in wild-type tomato plants. As chilling lasted, the decline was exaggerated in SBPase antisense plants but was relieved in SBPase sense plants, implying that overexpressing SBPase could maintain relatively higher ΦPSII, which could help to restore part of reduced photosynthesis caused by chilling stress. Additionally, high levels of carbohydrate in tomato plants with increased SBPase activity as observed in this study may also contribute to improved chilling tolerance, as it has been shown that high accumulation of carbohydrate ameliorates chilling sensitivity in plants^40^,^41^. All these pieces of evidence support a role of high levels of SBPase activity in improving chilling tolerance in tomato plants.

In summary, changes in SBPase activity affects plant growth and development by altering photosynthetic capacity and carbohydrate accumulation. Improved chilling tolerance in tomato plants by enhanced SBPase activity can be partially ascribed to increased carbohydrate accumulation, higher RuBP regeneration rate and improved quantum efficiency of photosystem II. Our work provides a case study that an individual enzyme in the Calvin cycle may be a useful target for genetic engineering to improve production and stress tolerance in crops.

## Materials and Methods

### Plant materials and chilling treatment

Seeds of wild-type tomato (*Solanum lycopersicum* L. cv. ‘L-402′) and transgenic T1 progeny plants were germinated for 3 days in dark at 25 °C on filter paper in petri dishes. Seedlings were then planted individually in 38-cm-diameter plastic pots containing peat, vermiculite, soil and manure (v/v/v/v = 3:3:3:1). All plants were maintained and received the same management in a greenhouse in Tai’an (36° 11′ 7″ N, 117° 7′ 12″ E), China, from February to July, with the day/night temperature regime being 20–30 °C/10–15 °C and the light level being 200–1000 μmol m^−2 ^s^−1^ depending on months and time of day. At the 20-leaf stage, post-maturation leaves (leaf no. 5 from base), mature fully expanded leaves (leaf no. 9, 13), new fully expanded leaves (leaf no. 17) and young expanding leaves (leaf no. 20) were used for measurements of development-dependent photosynthetic rates, SBPase activity and *SlSBPase* expression level. For analysis of *SlSBPase* expression pattern, fully expanded leaves, two-week old fruits, stems and roots were sampled in tomato plants. Fully expanded leaves (leaf no. 13–15) were used to measure diurnal changes of photosynthetic rates, SBPase activity and *SlSBPase* expression level on three consecutive sunny days.

To investigate the role of SBPase in chilling tolerance, a group of wild-type and transgenic tomato plants were moved to a growth chamber at the 12-leaf stage for acclimation at 25/22 °C day/night at the light level of 400 μmol m^−2 ^s^−1^ with a photoperiod of 14 h for a week. Then, three sets of wild-type tomato plants, SBPase sense tomato plants and SBPase antisense tomato plants were subjected to chilling stress at 5 °C for a period of 12 h. Photosynthetic rates and fluorescent parameters were measured at 0, 2, 6 and 12 h. Following measurement of each time point, leaf samples were harvested for SBPase activity assay and the analysis of electrolyte leakage.

### Isolation of *SlSBPase* gene from tomato leaf

Total RNA was extracted from young fully expanded leaves in tomato plants using Trizol (TaKaRa, Japan). First strand cDNA was synthesized according to standard protocol (PrimeScript^®^ reverse transcriptase protocol, TaKaRa, Japan). A total of 10 μl mixture of 50 pmol Oligo dT primer, 10 mM dNTP mixture, 1μg total RNA and RNase free dH_2_O was heated at 65 °C for 5 min and immediately cooled on ice. Then, a total volume of 20 μl mixture containing RNA/primer mixture, PrimeScript buffer, RNase inhibitor, PrimeScript reverse transcriptase and RNase free dH_2_O was incubated at 42 °C for 60 min. The resulting cDNA mixture was used as a template for the following PCR. The full-length cDNA sequence of *SlSBPase* was amplified using gene-specific primers SBP-RT-F and SBP-RT-R (see Supplementary Table S1). PCR was performed with HS PrimeSTAR^®^ DNA polymerase (TaKaRa). The corresponding cDNA fragments were separated by gel electrophoresis (1% agarose) and extracted using the DNA purification kit (DP214, TIANGEN, Beijing, China). These fragments were subsequently ligated into pMD-18T vector (TaKaRa) and transformed into DH5α competent cells (TIANGEN) and verified by sequencing. All subsequent constructs were made using this clone as template.

#### *Agrobacterium tumefaciens*-mediated genetic transformation of tomato plants

The full-length coding sequence and a 563-bp DNA fragment of *SlSBPase* were amplified using primers labeled as SBP-ORF-F(R) and SBP-An-F(R) in Supplementary Table S1, respectively. Fragment containing the open reading frame sequence were cloned into the plant expression vector pBI121 under the control of CaMV35S promoter to generate sense constructs [pBI-SBP(+)], and the 563-bp DNA fragment was used to generate antisense constructs [pBI-SBP(−)]. The constructs were then introduced into *Agrobacterium tumefaciens* LBA4404 and then transformed into tomato with cotyledon as explants as described by Dan *et al.*^42^. Shoots were regenerated on selective medium containing kanamycin (50 mg L^−1^). Rooted kanamycin-resistant primary transformants (T0 generation) were transferred to soil and allowed to self-fertilize. Kanamycin-resistant plants were also confirmed for the presence of SlSBPase gene by PCR. The seeds from T0 plants were sown and the resulting plants (T1 generation) were used for further analysis.

#### Analysis of transcript abundance by quantitative real-time PCR

The RNA samples from different tissues and leaves were used for cDNA synthesis by PrimeScript^®^ reverse transcriptase following standard protocols. Quantitative real-time PCR (qRT-PCR) was carried out using SYBR^®^ Premix Ex TaqTM (TaKaRa) according to manufacturer’s instructions. Tomato actin gene was used as an internal constitutively expressed control. Each real-time PCR reaction was performed in 25 μl final volume on iQ5 Multicolor Real-Time PCR Detection System (BIO-RAD, USA) under the following program: 1 cycle of 30 s at 95 °C, followed by 40 cycles of 5 s at 95 °C, 30 s at 60 °C.

#### SDS-PAGE and immunological analysis

The coding region of *SlSBPase* was amplified using primers labeled as SBP-pET-F and SBP-pET-R in Supplementary Table S1. The amplified fragment was subcloned into the pET-30a (+) vector between the *Bam*HI and *Sal*I sites. The pET-SlSBPase were transformed to *Escherichia coli* BL21, and expression was induced by 0.4 mM IPTG for 6 h at 37 °C. Cells were harvested and separated by SDS-PAGE using 12% separate gels and 5% concentrated gels. The strong induced fusion protein bands were collected into phosphate buffer solution (PBS) and were used to immunize BALB/c male mice (6-week old) with 4 subcutaneous injections at one-week interval to obtain primary antibody. Blood was collected and the sera were tested with an enzyme-linked immunosorbent assay. For protein-blot analysis, tomato tissues were ground in SDS sample buffer (1: 6w/v) and protein concentrations were determined. Proteins (25 μg per lane) were separated by SDS-PAGE, blotted onto PVDF membranes (Millipore) and probed with anti-SlSBPase serum (1: 500). Serum cross-reacting proteins were visualized using a horseradish peroxidase (HRP)-linked secondary antibody at a dilution of 1: 500 (Santa Cruz Biotechnology, Inc.).

### SBPase activity assay

SBPase activity assay was performed as described by Harrison *et al.*^14^. Samples were thoroughly ground in liquid nitrogen and then transferred to 1 ml extraction buffer [50 mM Hepes, pH 8.2; 5 mM MgCl_2_; 1 mM EDTA; 1 mM EGTA; 10% glycerol; 2 mM benzamidine; 2 mM amino caproic acid; 0.5 mM phenylmethylsulfonyluoride (PMSF); 10 mM dithiothreitol (DTT)]. The supernatant after centrifuging was desalted before being used for enzyme assay. The supernatant was subjected to a NAP-10 column (GE Healthcare Life Sciences, Pittsburgh, USA) equilibrated with desalting buffer (extraction buffer ommiting Triton X-100). Proteins were eluted with 1.2 ml desalting buffer and then used for measuring SBPase activity. For the assay, to start the reaction, 20 μl of protein sample was added to 80 μl of assay buffer (50 mM Tris, pH 8.2; 15 mM MgCl_2_; 1.5 mM EDTA; 10 mM DTT; 2 mM SBP (Organix UK, University of Essex, Essex, UK) and incubated at 25 °C for 5 min. The reaction was stopped by the addition of 50 μl of 1 M perchloric acid. The samples were then centrifuged for 5 min and the supernatant assayed for phosphate. 0.5 mM samples (50 μl) and phosphate standards (0–0.5 mM NaH_2_PO_4_) were incubated with 850 μl molybdate solution (0.3% ammonium molybdate in 0.55 M H_2_SO_4_) for 10 min at room temperature. Malachite green (0.035% malachite green, 0.35% polyvinyl alcohol) was added (150 μl) and the samples incubated for a further 45 min at room temperature before measuring the absorbance at 620 nm with UV-visible spectrophotometer (UV-2450, Shimadzu Corporation, Japan).

### Determination of photosynthesis and quantum efficiency of photosystem II

Photosynthetic rates were measured using an open gas exchange system (CIRAS-1, PP-Systems, Hitchin, Herts., UK) under ambient light and CO_2_ in greenhouse conditions. Measurements were performed on post-maturation leaves (leaf no. 5 from base), mature fully expanded leaves (leaf no. 9, 13), new fully expanded leaves (leaf no. 17) and young expanding leaves (leaf no. 20) at 10:30–11:30 a.m. on a sunny day. The diurnal changes of photosynthesis were measured on fully expanded leaves (leaf no. 13–15) beginning at 6:00 a.m. and finishing at 6:00 p.m. at 2-h intervals on a sunny day. Simultaneously, photon flux density (PFD) and air temperature (Ta) at each time point over the day were measured by a photometer (LI-18P, LI-COR Company, USA) and a thermometer, respectively. Photosynthesis of transgenic tomato plants was measured on the fully expanded leaves (leaf no. 8, 9) at the 12-leaf stage at 10:30–11:30 a.m. on a sunny day.

Tomato plants were dark adapted for 30 min and the minimal fluorescence from a dark-adapted leaf (Fo) was measured and following a saturating pulse, the maximal fluorescence from a dark-adapted leaf (Fm) was obtained, allowing us to calculate the maximum quantum efficiency (Fv/Fm). The operating quantum yield of PSII (ΦPSII) in illumination was measured after 15 min of illumination with a modulated chlorophyll fluorescence spectrometer (FMS-2, Hansatech, UN).

### Calculation of RuBP regeneration rate

RuBP regeneration rates of tomato plants under chilling stress were derived from *A*/*C*i response curves according to equations of von Caemmerer and Farquhar^43^. *A* to *C*i response curves were obtained by measuring carbon assimilation rates of transgenic plants and wild-type plants using a gas exchange system (CIRAS-2, PP-Systems, Hitchin, Herts., UK) under a series of CO_2_ concentrations (50–1400 μmol mol^−1^) and suturing light. The data from the *A*/*C*i curves were used for the calculation of RuBP regeneration rates.

### Carbohydrate analysis

At the 12-leaf stage, same leaves used for measurements of photosynthesis in greenhouse-grown wild-type and transgenic tomato plants were harvested at the end of day for carbohydrate analysis. The carbohydrate levels were determined as described by Maeda *et al.*^44^ and Stitt *et al.*^45^.

### Electrolyte leakage assay

Leaves were harvested at 0, 2, 6 and 12 time points during chilling stress for electrolyte leakage assay. Electrolyte leakage was measured according to Ishitani *et al.*^31^.

### Growth analysis

At the 12-leaf stage, plan height, stem diameter, leaf length and leaf width were measured in wild-type and transgenic tomato plants. The flowering time was recorded as number of days after planting.

### Statistical analysis

All of the experiments were repeated at least three times, and the values presented are means ± SDs. Duncan’s multiple range test was performed to compare the difference among treatments. An asterisk in figures and tables indicates significant differences relative to the WT at *P* < 0.05 (** for *P* < 0.01).

## Additional Information

**How to cite this article**: Ding, F. *et al.* Changes in SBPase activity influence photosynthetic capacity, growth, and tolerance to chilling stress in transgenic tomato plants. *Sci. Rep.* **6**, 32741; doi: 10.1038/srep32741 (2016).

## Supplementary Material

Supplementary Information

## Figures and Tables

**Table 1:** Effects of changes in SBPase activity on plant height, stem diameter, leaf length, leaf width, leaf length/width ratios and flowering time in SBPase sense and antisense tomato plants.

Lines	Plant height (cm)	Stem diameter (cm)	Leaf length (cm)	Leaf width (cm)	Length/width ratio	Onset of flowering
WT	53.1 ± 1.73	0.65 ± 0.05	8.2 ± 0.09	3.9 ± 0.08	2.1 ± 0.04	51 ± 0.8
T1-6	53.1 ± 1.77	0.71 ± 0.05	8.2 ± 0.30	4.2 ± 0.23	2.0 ± 0.06	47 ± 1.5
T1-17	53.0 ± 2.31	0.81 ± 0.03^**^	8.0 ± 0.34	5.6 ± 0.27^**^	1.4 ± 0.09^**^	44 ± 1.2^*^
T1-3	52.3 ± 3.15	0.91 ± 0.10^**^	8.6 ± 0.33^*^	6.4 ± 0.31^**^	1.4 ± 0.09^**^	45 ± 1.3^*^
T1′-9	49.2 ± 2.35	0.58 ± 0.05	7.1 ± 0.50^*^	2.9 ± 0.10^**^	2.6 ± 0.20^*^	55 ± 1.9
T1′-10	48.4 ± 0.69^*^	0.52 ± 0.07^*^	7.1 ± 0.40^*^	2.8 ± 0.10^**^	2.5 ± 0.20^*^	55 ± 1.0^*^
T1′-33	49.0 ± 0.50^*^	0.49 ± 0.02^**^	5.6 ± 0.30^**^	2.1 ± 0.20^**^	2.7 ± 0.30^*^	57 ± 0.6^*^

All measurements were made on the 12-leaf wild-type and T1 progeny of transgenic tomato plants. Flowering time was recorded as number of days after planting. Each value is the mean ± SD (n > 6). Asterisks indicate significant differences between WT and transgenic plants (* for *P* < 0.05; ** for *P* < 0.01).
