# Supplementary material for: Changes in SBPase activity influence photosynthetic capacity, growth, and tolerance to chilling stress in transgenic tomato plants
Source: Sci Rep. 2016 Sep 2;6:32741. doi: 10.1038/srep32741 (PMC5009361; doi:10.1038/srep32741)
Supplement: Supplementary Information [file srep32741-s1.doc]

**Changes in SBPase activity influence photosynthetic capacity, growth, and tolerance to chilling stress in transgenic tomato plants**

**Fei Ding^1,†^, Meiling Wang^2,†^, Shuoxin Zhang^1,*^ & Xizhen Ai^2,*^**

^1^College of Forestry, Northwest A&F University, 3 Taicheng Rd., Yangling, Shaanxi 712100, China. ^2^State Key Laboratory of Crop Biology, College of Horticulture Science and Engineering, Shandong Agricultural University, 61 Daizong St., Tai’an, Shandong 271018, China.

^†^These authors contributed equally to this work.

**^*^**Correspondence and requests for materials should be addressed to S.Z. (email: sxzhang@nwsuaf.edu.cn) or X.A. (email: axz@sdau.edu.cn)

**Supplementary Table S1. Primers used in this study.** Restriction enzyme sites are in bold.

| Primer name | Primer sequence (5' 3') |
| --- | --- |
| SBP-RT-F | AATGGAGACTGGTGTTACATGT |
| SBP-RT-R | TTCTCAAGCCTCAAACAGCAG |
| SBP-ORF-F | CGC**GGATCC**AAAATGGAGACTGGTGTT |
| SBP-ORF-R | ATGC**GTCGAC**TTCTCAAGCCTCAAACAG |
| SBP-An-F | AGGC**GTCGAC**TTCTTTTGGTGATGAAC |
| SBP-An-R | CGC**GGATCC**TGAATATACCTTTCTCCT |
| SBP-pET-F | CGC**GGATCC**AAAATGGAGACTGGTGTT |
| SBP-pET-R | ATGC**GTCGAC**TTCTCAAGCCTCAAACAG |
| SBP-qPCR-F | CGTGACATCTCCAACAGCTAAGG |
| SBP-qPCR-R | CATCGCTGCTGTAACCTCCAG |
| Actin-F | ATGTATGTTGCTATTCAGGCTGTG |
| Actin-R | TAACCCTCGTAGATAGGGACAG |
| rbcL-F | GCTGTATTTGCTAGAGAATTGG |
| rbcL-R | GGTGGATGTGAAGAAGTAGACC |
| rbcS-F | AGCATGGTCGCACCCTTC |
| rbcS-R | CCACACCTGCATGCATCT |
| FBPase-F | CAGCTCCAGCACAGATCAAG |
| FBPase-R | TGCAGGCTAGTGATATGCTCG |
| FBA-F | AGTACTACGAGGCTGGTGCT |
| FBA-R | CCATTGGCGTTATCATTGAT |
| TK-F | ACTGGTTTAATCGTGACCGAT |
| TK-R | TGACTTCAACACCAGGTGTCTC |
| GAPDH-F | GAAGCATCATGAGCTCAAGGT |
| GAPDH-R | CCTTCAAATGAGCTGCAGCT |


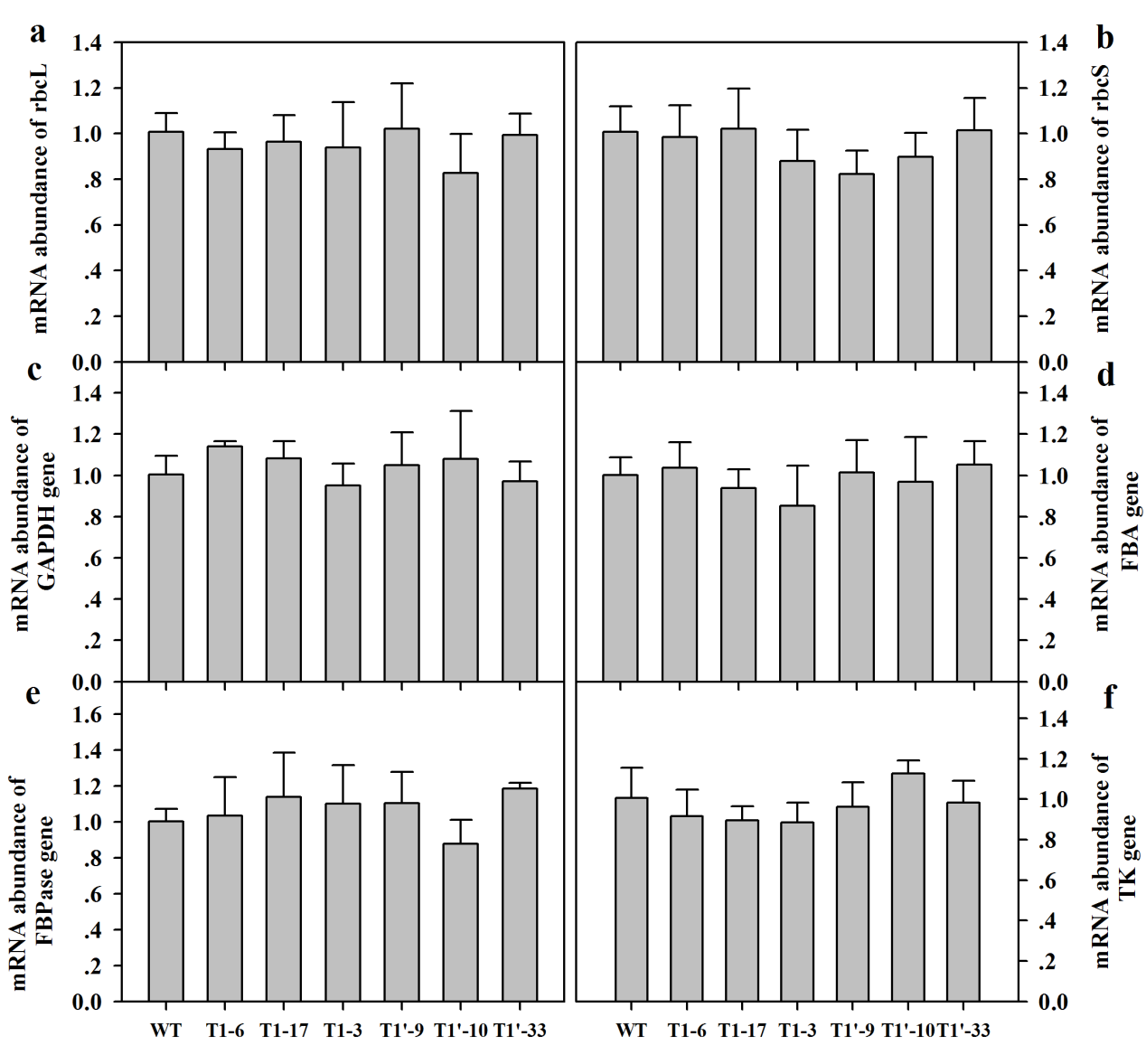


**Supplementary Fig. S1. Transcript abundance of enzyme-coding genes in the Calvin cycle investigated by quantitative real-time PCR.** The results are the means ± SDs (n = 3)
